# Profiling B and T cell immune responses to co-infection of *Mycobacterium tuberculosis* and hookworm in humans

**DOI:** 10.1186/s40249-015-0046-0

**Published:** 2015-05-04

**Authors:** Xin-Xu Li, Jia-Xu Chen, Li-Xia Wang, Jun Sun, Shao-Hong Chen, Jun-Hu Chen, Xiao-Yan Zhang, Xiao-Nong Zhou

**Affiliations:** National Institute of Parasitic Diseases, Chinese Center for Disease Control and Prevention, Key Laboratory of Parasite and Vector Biology, Ministry of Health, WHO Collaborating Centre for Malaria, Schistosomiasis and Filariasis, 207 Rui Jin Er Road, Huangpu District, Shanghai, 200025 PR China (PRC); National Center for Tuberculosis Control and Prevention, Chinese Center for Disease Control and Prevention, Beijing, 102206 PR China; Shanghai Public Health Clinical Center, Key Laboratory of Medical Molecular Virology of Ministry of Education, Institutes of Biomedical Sciences, Fudan University, Shanghai, 201508 PR China

**Keywords:** Co-infection, *Mycobacterium tuberculosis*, Hookworm, Immune response

## Abstract

**Background:**

Humoral and cellular immune responses play protective roles against *Mycobacterium tuberculosis* (MTB) infection. However, hookworm infection decreases the immune response to hookworm and bystander antigens. Currently, immune responses to co-infection of MTB and hookworm are still unknown, although co-infection has been one of the public health problems in co-endemic areas of pulmonary tuberculosis (PTB) and hookworm disease. Therefore, it is essential to evaluate B and T cell immune responses to the co-infection.

**Methods:**

Seventeen PTB cases co-infected with hookworm, 26 PTB cases, 15 patients with hookworm infection, and 24 healthy controls without PTB or hookworm infection were enrolled in the study. Expressions of CD3, CD4, CD8, CD10, CD19, CD20, CD21, CD25, CD27, CD38, FoxP3, and PD-1 were assessed on B and T cell subsets using multicolor flow cytometry.

**Results:**

For the B cell (CD19^+^) subsets, naïve B cells (CD10^−^CD27^−^CD21^+^CD20^+^), plasma cells (CD10^−^CD27^+^CD21^−^CD20^−^), and tissue-like memory B cells (CD10^−^CD27^−^CD21^−^CD20^+^) had higher proportions, whilst resting memory B cells (CD10^−^CD27^+^CD21^+^CD20^+^) had lower proportions in the group co-infected with MTB and hookworm as compared to other groups. Frequencies of activated memory B cells (CD10^−^CD27^+^CD21^−^CD20^+^) did not differ among the four groups. For the T cell (CD3^+^) subsets, frequencies of regulatory T cells (CD4^+^CD25^+^Foxp3^+^) and exhausted CD4^+^ and CD8^+^ T cells (CD4^+^PD-1^+^ and CD8^+^PD-1^+^) were higher, and frequencies of activated CD4^+^ and CD8^+^ T cells (CD4^+^CD38^+^ and CD8^+^CD38^+^) were lower in the co-infected group as compared to the other groups.

**Conclusion:**

The change patterns of the cell profile of circulating lymphocytes were indentified in human co-infection of MTB and hookworm, which might indicate that the humoral and cellular immune responses are more suppressed.

**Electronic supplementary material:**

The online version of this article (doi:10.1186/s40249-015-0046-0) contains supplementary material, which is available to authorized users.

## Multilingual abstracts

Please see Additional file 1 for translations of the abstract into the six official working languages of the United Nations.

## Background

Tuberculosis (TB) and hookworm infection are among the most important public health problems worldwide. In 2011, there were an estimated 8.7 million new TB cases and 1.4 million people died from the disease globally, with the burden of TB geographically highest in Asia and Africa [1]. The World Health Organization (WHO) also reported that hookworm infection afflicted an estimated 740 million people in the developing nations of the tropics in 2003, and the largest numbers of cases occurred in impoverished rural areas of Sub-Saharan Africa, Latin America, Southeast Asia, and China [2]. In addition to being two independent major health problems, the observed associations between TB and hookworm infection are important, although there have been little related studies conducted worldwide [3]. For example, the prevalence of hookworm infection among TB cases was about 6%–11%, while TB prevalence among patients with hookworm infection was near 17% in some East African countries [4-6]. Therefore, it is essential to profile the human co-infection of *Mycobacterium tuberculosis* (MTB) and hookworm.

MTB is a facultative intracellular pathogen. The effective cell-mediated immune response to MTB infection, involving mainly the CD4^+^ and CD8^+^ T cell subsets, plays an essential role in the pathogenesis of TB [7,8]. Despite this, emerging evidence suggests that B cells and humoral immunity can also modulate the immune response to MTB infection [9,10]. Unlike MTB infection, which is phagocytosed by resident alveolar macrophages and tissue dendritic cells in the lung and replicates inside these cells [11], hookworm infection presents the host with an extensive diversity of antigenic challenges, immune stimulation, and immune modulation (including humoral and cellular responses) during various stages, from skin invasion, to transit through lung tissues, to arrival in the gut and penetration of its mucosa [12]. Many studies have confirmed that hookworm infection decreases the ability of the immune system to respond to hookworm and bystander antigens, as evidenced by decreased lymphocyte responses in hookworm-infected humans [13-15]. However, the immune system response to co-infection of MTB and hookworm in humans has still not been clarified.

In order to evaluate B and T cell immune responses to co-infection of MTB and hookworm, this study compared alterations of B and T cell subsets, expressions of whose markers were analyzed by flow cytometry [16] in pulmonary TB (PTB) cases with and without hookworm infection, patients only with hookworm infection, and healthy controls without PTB or hookworm infection.

## Methods

### Study population

The study was conducted in Gushi County of Henan province, which is an agricultural county that lies in the center of China. The study was conducted between July and September 2012 [17]. Seventeen PTB cases co-infected with hookworm (TB + HW group), 26 PTB cases without hookworm infection (TB group), 15 patients only with hookworm infection (HW group), and 24 healthy controls without PTB or hookworm infection (HC group) were enrolled in the study. All PTB cases were selected from the TB surveillance system, diagnosed according to the diagnostic criteria of the National Tuberculosis Program (criteria includes three sputum smear examinations, chest imaging, and clinical symptoms) [18]. Two stool specimens were collected for the diagnosis of the hookworm infection and three smears of each stool specimen were examined by the modified Kato-Katz thick smear technique (a semi-quantitative stool examination technique for detection of helminthic ova) [19]. The egg count for hookworm was not assessed. Apart from hookworm, there were no other helminth infections in participants. No participant received any anti-parasitic treatment against hookworm before blood collection. There were no statistical differences between the ages of the participants from all four groups: TB + HW (median age 60 years), TB (median age 61 years), HW (median age 65 years), and HC (median age 62 years). All PTB cases received anti-TB treatment as PTB cases are treated immediately once they are diagnosed based on the national guidelines in China. The main regimen of anti-MTB treatment is the combination of isoniazid, rifampicin, pyrazinamide, ethambutol, and streptomycin, or their derivatives [18]. The duration of anti-TB treatment was similar between the TB + HW group (median 4.5 months) and the TB group (median 4.4 months) (see Table 1). General medical checkups confirmed that participants had no organic or immune system diseases. Participants from the HW and HC groups were confirmed non-TB by sputum smear examination. All participants were HIV negative.

**Table 1:** **Characteristics of study participants [Median (IQR)]**

**Group**	**N**	**Sex (n)**		**Age (years)**	**Duration of anti-TB treatment (months)**
		**Male**	**Female**		
HC	24	14	10	62 (48–70)	NA
HW	15	10	5	65 (56–70)	NA
TB	26	15	11	61 (44–67)	4.4 (2.5–5.3)
TB + HW	17	9	8	60 (50–72)	4.5 (3.0–5.4)

IQR: interquartile range; HC: healthy controls without PTB or hookworm infection; HW: patients only with hookworm infection; TB: PTB cases without hookworm infection; TB + HW: PTB cases co-infected with hookworm; NA: not applicable.

### Ethical statement

The study was evaluated and approved by the Ethics Review Committee of the National Institute of Parasitic Diseases, Chinese Center for Disease Control and Prevention. All participants gave their written informed consents prior to the study commencing. At the completion of the study, anti-parasitic treatment was offered at no charge to all participants with positive hookworm infection, in accordance with the local treatment guidelines.

### Cells preparation

EDTA-treated whole blood was obtained from all participants and sent to the Key Laboratory of Medical Molecular Virology of Ministry of Education, Institutes of Biomedical Sciences, Fudan University (Shanghai, China) within eight hours. Peripheral blood mononuclear cells (PBMCs) were isolated from EDTA-treated whole blood by Ficoll-Hypaque (Sigma Chemical Co., St Louis, MO, USA) density gradient centrifugation.

### Flow cytometric analysis

The freshly isolated PBMCs were washed with phosphate-buffered saline (PBS)-2% fetal bovine serum and stained with surface antibodies (CD19-FITC, CD10-PE, CD20-PerCP-Cy5-5, CD21-APC, CD27-PE-Cy7, CD3-FITC, CD4-PE-Cy7, CD8-Pacific Blue, CD25-PE, FoxP3-APC, CD38-APC-Cy7, PD-1-PerCP-Cy5-5) for 30 minutes at 4°C in the dark. The clones used for each antibody were HIB19, eBioCB-CALLA, IH7, HB5, 0323, OKT3, SK3, SK1, Bc96, PCH101, HIT2, and EBioJ105, respectively (see Table 2). Cells were then washed, fixed, and permeabilized using the Foxp3/Transcription Factor Staining Buffer Set kit (eBioscience, San Diego, CA, USA), according to its instructions. Following fixation, the cells were washed twice in the perm buffer and incubated for 30 minutes at 4°C, with anti-human Foxp3 antibodies conjugated to allophycocyanin (APC). Following staining, the cells were washed, fixed (PBS containing 1% paraformaldehyde), and stored at 4°C until analysis (within 24 hours) in a modified FACSAria™ flow cytometer (BD Immunocytometry Systems, BD Bioscience, San Jose, CA, USA). All surface antibodies were purchased from eBioscience (San Diego, CA, USA). Data acquisition and analysis was performed using the BD FACSDiva™ software (BD Bioscience, San Jose, CA, USA).

**Table 2:** **Antibodies used for flow cytometry**

**Antigen**	**Clone**	**Color**
CD3	OKT3	FITC
CD4	SK3	PE-Cy7
CD8	SK1	Pacific Blue
CD10	eBioCB-CALLA	PE
CD19	HIB19	FITC
CD20	IH7	PerCP-Cy5-5
CD21	HB5	APC
CD25	Bc96	PE
CD27	0323	PE-Cy7
CD38	HIT2	APC-Cy7
FoxP3	PCH101	APC
PD-1	EBioJ105	PerCP-Cy5-5

APC: allophycocyanin; FITC: fluorescein isothiocyanate; PE: phycoerythrin; PerCP: peridinin chlorophyll protein.

### Evaluation of B cell subsets

**Figure 1:**
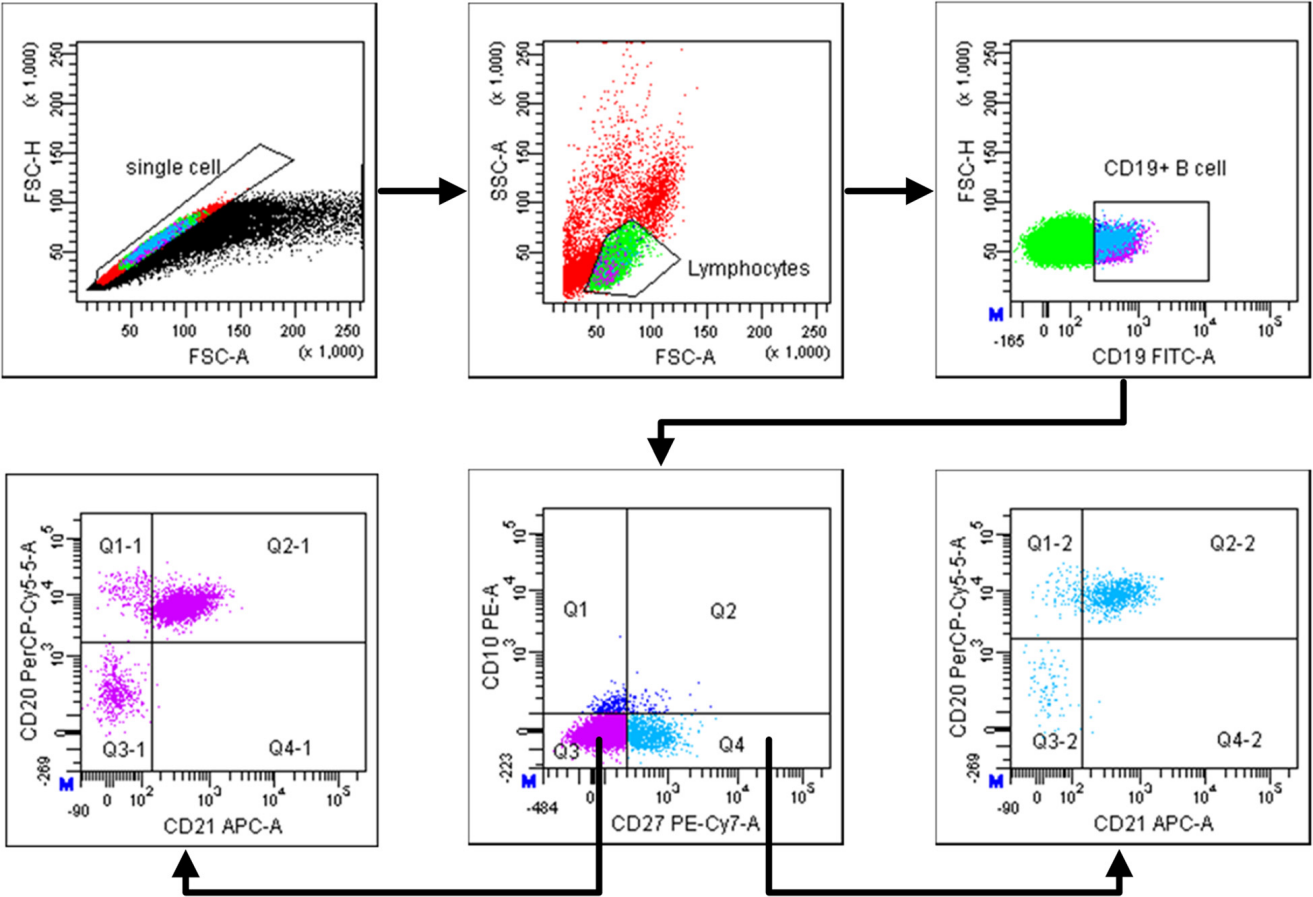
Representative flow cytometry gating strategy for identification of B cell (CD19^+^) subsets: naïve B cells (CD10^−^CD27^−^CD21^+^CD20^+^), tissue-like memory B cells (CD10^−^CD27^−^CD21^−^CD20^+^), activated memory B cells (CD10^−^CD27^+^CD21^−^CD20^+^), resting memory B cells (CD10^−^CD27^+^CD21^+^CD20^+^), and plasma cells (CD10^−^CD27^+^CD21^−^CD20^−^) in peripheral blood mononuclear cells.

Gating strategies were set to evaluate B cell subsets (see Figure 1). Peripheral B cell is only necessary to gate on CD19^+^ cells that also co-expressed CD20 [20]. CD10 expression defines two populations of B cell, a minor mature population that co-expresses CD27 and an immature CD27^−^ population [21]. Naïve B cells are defined as CD19^+^CD10^−^CD27^−^CD21^+^CD20^+^ B cells, and resting memory B cells are defined as CD19^+^CD10^−^CD27^+^CD21^+^CD20^+^ B cells [22]. Mature activated memory B cells are identified by the B cell surface marker CD19^+^CD10^−^CD27^+^CD21^−^CD20^+^, and tissue-like memory B cells are identified by marker CD19^+^CD10^−^CD27^−^CD21^−^CD20^+^ [22]. Plasma cells are identified by marker CD19^+^CD10^−^CD27^+^CD21^−^CD20^−^ [23]. Definitions of B cell subsets are presented in Table 3.

**Table 3:** **Definitions of B cell subsets**

**Parameter**	**Subset**
CD19^+^	B cell
CD19^+^CD10^+^CD27^−^	Immature B cell
CD19^+^CD10^−^CD27^−^CD21^+^CD20^+^	Naïve B cell
CD19^+^CD10^−^CD27^−^CD21^−^CD20^+^	Tissue-like memory B cell
CD19^+^CD10^−^CD27^+^CD21^−^CD20^+^	Activated memory B cell
CD19^+^CD10^−^CD27^+^CD21^+^CD20^+^	Resting memory B cell
CD19^+^CD10^−^CD27^+^CD21^−^CD20^−^	Plasma cell

### Evaluation of T cell subsets

**Figure 2:**
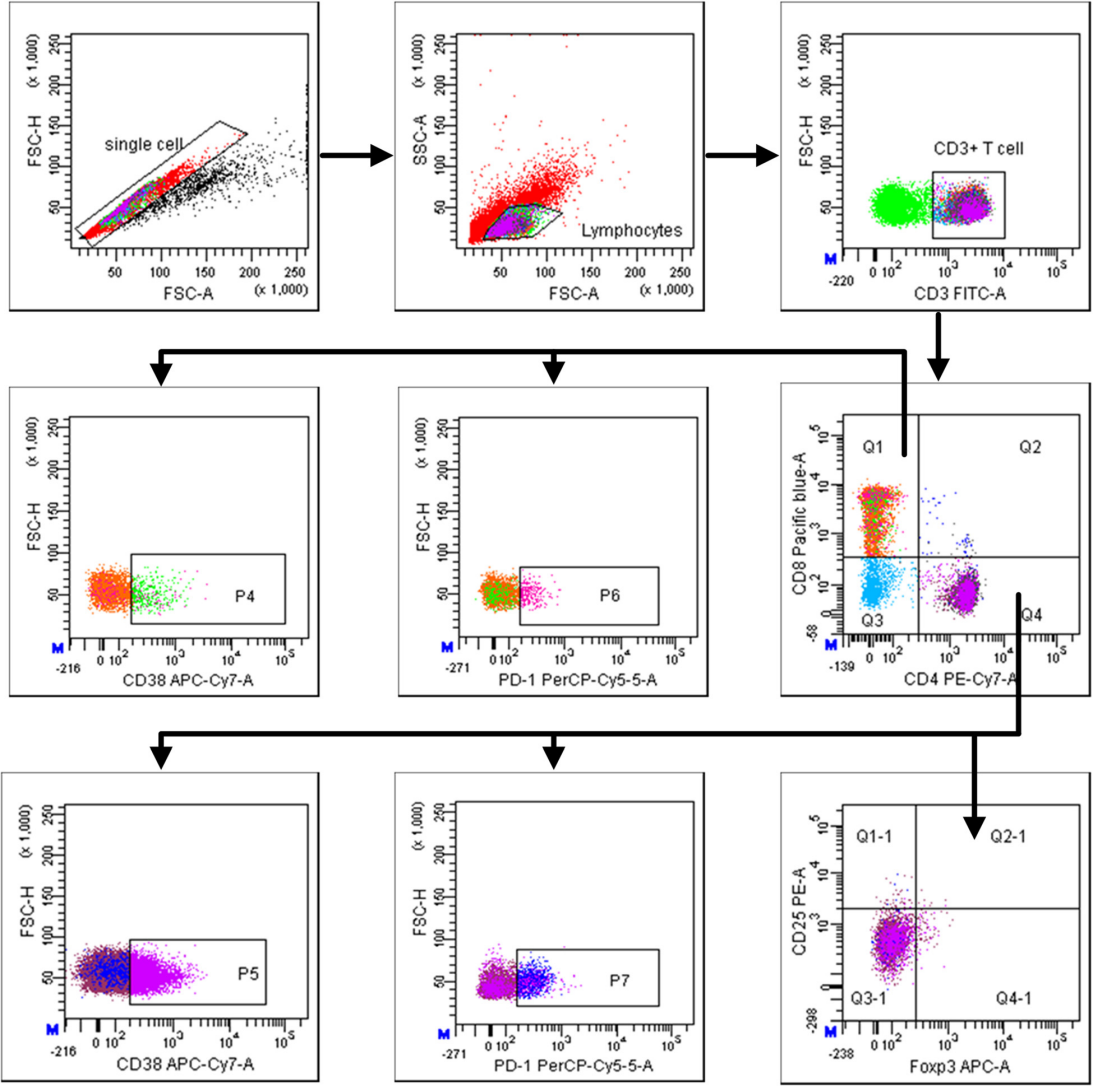
Representative flow cytometry gating strategy for identification of T cell (CD3^+^) subsets: regulatory T cells (CD4^+^CD25^+^Foxp3^+^), activated CD4^+^ T cells (CD4^+^CD38^+^), exhausted CD4^+^ T cells (CD4^+^PD-1^+^), activated CD8^+^ T cells (CD8^+^CD38^+^), and exhausted CD8^+^ T cells (CD8^+^PD-1^+^) in peripheral blood mononuclear cells.

Gating strategies were set to evaluate T cell subsets (see Figure 2). Peripheral T cell is necessary to gate on CD3^+^ cells because CD3 conformation is crucial for T cell signaling [24]. It is well known that expression of CD4 or CD8 on T cells is pivotal in defining them as T helper or T cytotoxic cells, respectively. In defining T cell subsets, we used the following nomenclature: regulatory T (Treg) cells (CD3^+^CD4^+^CD25^+^Foxp3^+^) [25], activated CD4^+^ T cells (CD3^+^CD4^+^CD38^+^) [26], exhausted CD4^+^ T cells (CD3^+^CD4^+^PD-1^+^) [27], activated CD8^+^ T cells (CD3^+^ CD8^+^CD38^+^) [26], and exhausted CD8^+^ T cells (CD3^+^CD8^+^PD-1^+^) [27]. Definitions of T cell subsets are presented in Table 4.

**Table 4:** **Definitions of T cell subsets**

**Parameter**	**Subset**
CD3^+^	T cell
CD3^+^CD4^+^	CD4^+^ T cell
CD3^+^CD8^+^	CD8^+^ T cell
CD3^+^CD4^+^CD25^+^Foxp3^+^	Regulatory T cell
CD3^+^CD4^+^CD38^+^	Activated CD4^+^ T cell
CD3^+^CD4^+^PD-1^+^	Exhausted CD4^+^ T cell
CD3^+^CD8^+^CD38^+^	Activated CD8^+^ T cell
CD3^+^CD8^+^PD-1^+^	Exhausted CD8^+^ T cell

### Statistical analysis

The immune parameters were measured using the percentage of each subpopulation in their respective populations, such as B cell (% of lymphocytes), immature B cell (% of B cell), T cell (% of lymphocytes), and Treg cell (% of CD4^+^ T cell) (see Tables 5 and 6). Characteristics of the study population and the different immune parameters were recorded as median (interquartile range [IQR]). Comparisons between groups were analyzed using the Wilcoxon Rank-Sum test, a non-parametric test. All statistical analyses and graphs were performed using R (Version 3.0.1, The R Foundation for Statistical Computing), a language and environment for statistical computing and graphics. Due to the small samples in this study, all two-tailed *p*-values were considered statistically significant when lower than 0.10 for capturing potential differences as much as possible, as *p*-values with the α < 0.10 critical value as a threshold perform better for statistical tests of small samples than other *p*-values [28].

**Table 5:** **Frequencies of B cell subsets [Median (IQR)]**

**Subset**	**HC**	**HW**	**TB**	**TB + HW**
B cell (%)	7.1 (4.7–8.9)	7.2 (3.5–7.7)	6.6 (4.6–7.6)	7.5 (5.8–8.4)
Naïve B cell (%)	63.8 (42.2–78.5)	71.9 (53.4–83.3)	63.4 (57.0–74.8)	78.8 (71.4–81.1)
Tissue-like memory B cell (%)	5.0 (4.1–6.8)	4.7 (3.8–6.0)	3.8 (2.3–4.9)	5.0 (4.2–6.1)
Activated memory B cell (%)	7.8 (5.1–10.0)	8.0 (7.3–9.0)	7.5 (5.2–10.6)	5.0 (4.8–8.6)
Resting memory B cell (%)	46.8 (29.5–69.1)	65.3 (43.2–70.4)	46.0 (37.8–54.8)	35.9 (33.6–43.4)
Plasma cell (%)	44.6 (16.6–66.2)	25.8 (18.0–48.1)	44.3 (38.2–53.9)	54.7 (47.8–60.2)

IQR: interquartile range; HC: healthy controls without PTB or hookworm infection; HW: patients only with hookworm infection; TB: PTB cases without hookworm infection; TB + HW: PTB cases co-infected with hookworm.

**Table 6:** **Frequencies of T cell subsets [Median (IQR)]**

**Subset**	**HC**	**HW**	**TB**	**TB + HW**
T cell (%)	65.5 (62.0–73.4)	60.9 (58.5–74.3)	65.2 (62.4–74.2)	72.2 (65.4–73.0)
Regulatory T cell (%)	0.9 (0.5–1.1)	0.9 (0.2–1.1)	0.7 (0.6–0.8)	1.3 (0.8–1.5)
Activated CD4^+^ T cell (%)	13.6 (8.5–16.4)	10.2 (8.9–17.7)	10.4 (8.0–12.6)	5.4 (3.9–10.8)
Exhausted CD4^+^ T cell (%)	8.4 (6.3–11.0)	9.4 (7.0–11.7)	7.8 (7.0–12.3)	11.4 (9.7–14.8)
Activated CD8^+^ T cell (%)	5.4 (4.4–8.0)	4.6 (4.1–6.1)	4.1 (3.5–6.6)	3.6 (2.7–5.2)
Exhausted CD8^+^ T cell (%)	9.4 (6.7–11.4)	10.8 (8.3–13.4)	7.6 (5.3–10.4)	11.2 (9.6–12.3)

IQR: interquartile range; HC: healthy controls without PTB or hookworm infection; HW: patients only with hookworm infection; TB: PTB cases without hookworm infection; TB + HW: PTB cases co-infected with hookworm.

## Results

### Alterations of B cell subset in PTB cases with hookworm infection

**Figure 3:**
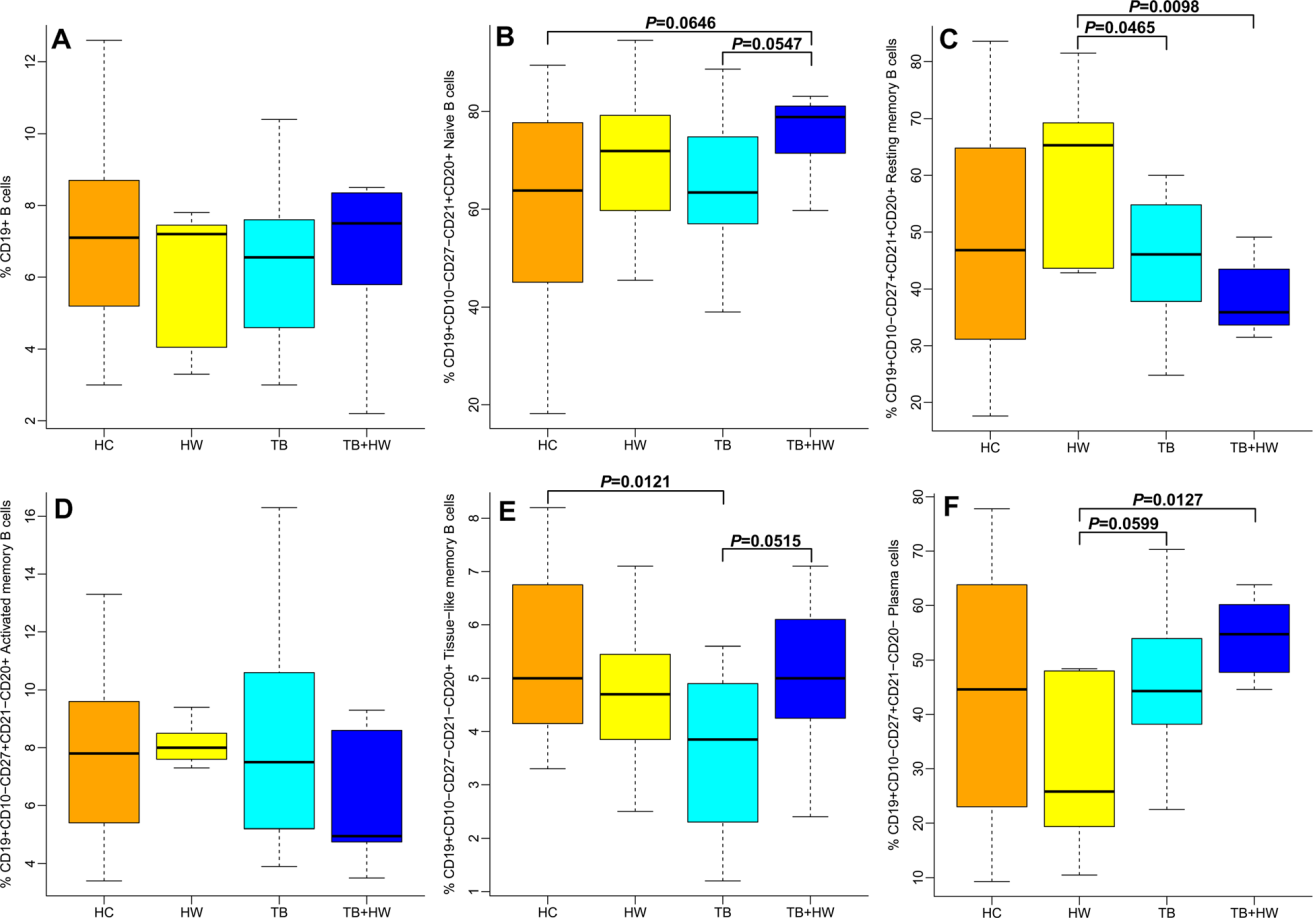
The expressions of B cell subsets. **A**: The proportions of B cells in healthy controls without PTB or hookworm infection (HC), patients only with hookworm infection (HW), PTB cases without hookworm infection (TB), and PTB cases co-infected with hookworm (TB + HW). **B**: The proportions of naïve B cells in HC, HW, TB, and TB + HW. **C**: The proportions of resting memory B cells in HC, HW, TB, and TB + HW. **D**: The proportions of activated memory B cells in HC, HW, TB, and TB + HW. **E**: The proportions of tissue-like memory B cells in HC, HW, TB, and TB + HW. **F**: The proportions of plasma cells in HC, HW, TB, and TB + HW.

We analyzed B cell frequencies and B cell subset frequencies to evaluate whether peripheral B cell subset compartment alters among the study groups (see Table 5 and Figure 3). Total percentages of peripheral B cells were similar among the four groups (see Figure 3A). The proportions of naïve B cells were found to be higher in the TB + HW group than in the HC group (*p* = 0.0646) and TB group (*p* = 0.0547) (see Figure 3B). Increased proportions of resting memory B cells were observed in the HW group compared with the TB group (*p* = 0.0465) and TB + HW group (*p* = 0.0098) (see Figure 3C). The proportions of activated memory B cells were also similar among the four groups (see Figure 3D). The percentages of tissue-like memory B cells were found to be lower in the TB group than in the HC group (*p* = 0.0121) and TB + HW group (*p* = 0.0515) (see Figure 3E), and decreased proportions of plasma cells were observed in the HW group as compared to the TB group (*p* = 0.0599) and TB + HW group (*p* = 0.0127) (see Figure 3F).

### Alterations of T cell subset in PTB cases with hookworm infection

**Figure 4:**
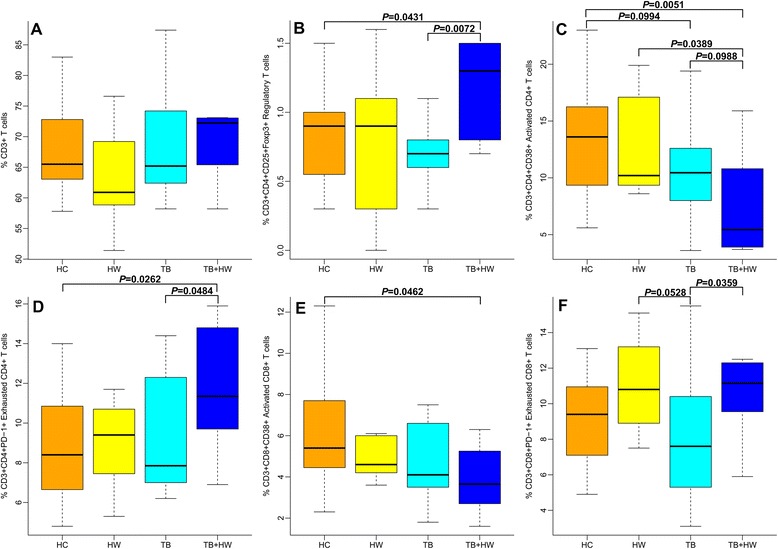
The expressions of T cell subsets. **A**: The proportions of T cells in healthy controls without PTB or hookworm infection (HC), patients only with hookworm infection (HW), PTB cases without hookworm infection (TB), and PTB cases co-infected with hookworm (TB + HW). **B**: The proportions of regulatory T cells in HC, HW, TB, and TB + HW. **C**: The proportions of activated CD4^+^ T cells in HC, HW, TB, and TB + HW. **D**: The proportions of exhausted CD4^+^ T cells in HC, HW, TB, and TB + HW. **E**: The proportions of activated CD8^+^ T cells in HC, HW, TB, and TB + HW. **F**: The proportions of exhausted CD8^+^ T cells in HC, HW, TB, and TB + HW.

We examined T cell frequencies and T cell subset frequencies to evaluate whether peripheral T cell subset compartment alters among the study groups (see Table 6 and Figure 4). Total frequencies of peripheral T cells were similar among the four groups (see Figure 4A). Higher percentages of Treg cells were detected in the TB + HW group than in the HC group (*p* = 0.0431) and TB group (*p* = 0.0072) (see Figure 4B). The TB + HW group showed lower frequencies of activated CD4^+^ T cells as compared to the HC group (*p* = 0.0051), HW group (*p* = 0.0389), and TB group (*p* = 0.0988), while the TB group also showed lower frequencies compared to the HC group (*p* = 0.0994) (see Figure 4C). In contrast, the TB + HW group showed higher frequencies of exhausted CD4^+^ T cells as compared to the HC group (*p* = 0.0262) and TB group (*p* = 0.0484) (see Figure 4D). The TB + HW group displayed lower proportions of activated CD8^+^ T cells as compared to the HC group (*p* = 0.0462) (see Figure 4E). The proportions of exhausted CD8^+^ T cells were lower in the TB group than in the HW group (*p* = 0.0528) and TB + HW group (*p* = 0.0359) (see Figure 4F).

## Discussion

Among parasitic infections that regulate or alter host immune responses, helminth infections often lead to systemic immune suppression or anergy [29]. As the important and common species of helminth, larval and adult hookworm release stage-specific antigenic molecules that induce antibody responses, eosinophilia, and florid intestinal inflammation via skin invasion, transit through lung tissues, and arrival in the gut and penetration of its mucosa [12]. However, there is no clear evidence that this offers the host any protection by significantly reducing larval and adult hookworm numbers [30]. In contrast, immunosuppression exists in patients with hookworm infection, and helminth infections including hookworm influence host immune responses to bystander antigens. For example, helminth infections are able to influence both the clinical outcome and the immune response of patients with cutaneous leishmaniasis [31]. Moreover, hookworm excretory/secretory products were observed to suppress intestinal pathology in a mouse model of colitis [32]. Up-to-date information on the immune responses to co-infection of MTB and hookworm in humans is unavailable.

Recent studies have suggested that B cells and humoral immunity can modulate host defense against various intracellular pathogens, including MTB, through a variety of interactions with the cellular immune response [9,10]. In this study, we found that higher percentages of naïve B cells and plasma cells existed in the TB + HW group compared to the TB or HW groups, although percentages of activated memory B cells did not differ among the groups. The primary antibody response is mediated by naïve B cells, and memory B cells mediate long-term protective immunity due to their capacity to generate secondary humoral responses [33]. After being activated following the receipt of signals through B-cell receptor, CD40, Toll-like receptor, and cytokine receptors, naïve B cells can enter a germinal center and then differentiate into either high-affinity plasma cells or memory B cells [33]. The results from our study indicated that naïve B cells became activated and then mostly differentiated into plasma cells in settings of co-infection of MTB and hookworm. However, we can hardly educe that the primary antibody response might be increased by MTB infection concurrent with hookworm infection in contrast with the secondary humoral response because boundaries between primary and secondary humoral responses blur for patients with chronic infectious diseases.

Tissue-like memory B cells expressed patterns of homing and inhibitory receptors and proliferated poorly in response to B cell stimuli, in which immunoglobulin diversities and replication histories have been shown to be lower [34]. Findings of this study showed that co-infection of MTB and hookworm increased proportions of tissue-like memory B cells in contrast with MTB infection, which demonstrated that activation of memory B cells and the humoral response against pathogens might be lower in PTB cases when they are co-infected with hookworm. Resting memory B cells are intrinsically programmed for enhanced survival and responsiveness to diverse stimuli compared to naïve B cells [35]. In this study, we found that co-infection of MTB and hookworm reduced frequencies of resting memory B cells compared to hookworm infection, which was further proof that the humoral response is more suppressed in patients with hookworm when they are co-infected with MTB.

The importance of CD4^+^ and CD8^+^ T cells for protection against TB is well known. After antigen presentation by accessory cells, CD4^+^ T cells are activated to produce IFN-γ, the prototypic Th1 cytokine that enhances the mycobactericidal capacity of macrophages [36,37], and CD8^+^ T cells also contribute to producing IFN-γ, lysing mycobacteria-infected macrophages, and killing MTB through a granule-dependent mechanism [37,38]. Defined by a poor effector function, sustained expression of inhibitory receptors and a transcriptional state distinct from that of functional effector or memory T cells, T cell exhaustion is a state of T cell dysfunction that arises during many chronic infections and cancer, which means that exhaustion prevents optimal control of infection and tumors [39]. In this study, we found different changes in the cell profile among the study groups: the percentages of activated CD4^+^ and CD8^+^ T cells were lower and exhausted CD4^+^ and CD8^+^ T cells were higher in the TB + HW group as compared to the other study groups, which suggests that T cell activation is weakened and T cell exhaustion is elevated in settings of co-infection of MTB and hookworm. Many studies have shown that hookworm antigens induce cell apoptosis by an intrinsic mitochondrial pathway, and hookworm tissue inhibitor of metalloproteases (Ac-TMP-1) induces generation of suppressor CD4^+^ and CD8^+^ T cells [40,41]. This points to the fact that hookworm infection depresses T cell immunity of PTB cases to a certain extent and leads to T cell dysfunction.

High levels of circulating Treg cells were found in patients with active PTB, compared to individuals with latent infection, with Treg cells inhibiting protective Th1 responses and facilitating mycobacterial replication and tissue damage [42]. Likewise, Treg cells may play an important role in hookworm-induced immunosuppression, contributing to the longevity of hookworm survival in infected people [43]. Moreover, a study demonstrated that Treg cells in human geohelminth (mainly hookworm) infection suppress immune responses to bystander antigen of mycobacteria [44]. In this study, we also found that co-infection of MTB and hookworm raised the proportions of Treg cells compared to MTB infection alone, which means that T cell-mediated immune response is more suppressed in PTB cases when they are co-infected with hookworm. Some studies indicated Treg cells control the size of the peripheral activated CD4^+^ T cell compartment and suppress cytotoxicity of CD8^+^ T cells [45,46], which is in agreement with findings about Treg cells and T cell exhaustion in this study.

A study found that coincident hookworm infection exerts a profound inhibitory effect on protective Th1 and Th17 responses in latent TB and may predispose toward the development of active TB in humans [47]. Although undergoing anti-TB treatment probably affects the immune responses to MTB infection [48], we also found that T cell immune response might be more suppressed by co-infection of MTB and hookworm in PTB cases with anti-TB treatment for about four months. This suggests that undergoing anti-TB treatment does not affect coincident hookworm infection inhibiting T cell immune response to MTB infection.

This study has a number of limitations. First, the sample size was small because participants were only recruited from patients with positive hookworm infection and their controls who all were included in a previous cross-sectional survey [17]. Second, participants were elderly because most young adults in the county leave for big cities for employment opportunities, which might have an influence on the immune responses to MTB or hookworm infections [49,50]. Lastly, durations and intensities of hookworm infection that might also have an influence on the immune responses were not evaluated. In view of these limitations, findings of this study should be interpreted carefully when generalized to the larger population and compared with results from other studies.

## Conclusion

Our study found that, in terms of co-infection with MTB and hookworm, for the B cell subsets, naïve B cells, plasma cells, and tissue-like memory B cells had higher proportions and resting memory B cells had lower proportions. For the T cell subsets, frequencies of Treg cells and exhausted CD4^+^ and CD8^+^ T cells were higher and frequencies of activated CD4^+^ and CD8^+^ T cells were lower. The change patterns of the cell profile of circulating lymphocytes were indentified in human co-infection of MTB and hookworm, which might indicate that the humoral and cellular immune responses are more suppressed.

## Additional file

Additional file 1:
**Multilingual abstracts in the six official working languages of the United Nations.**
