# Supplementary material for: Profiling B and T cell immune responses to co-infection of Mycobacterium tuberculosis and hookworm in humans
Source: Infect Dis Poverty. 2015 May 4;4:20. doi: 10.1186/s40249-015-0046-0 (PMC4423492; doi:10.1186/s40249-015-0046-0)
Supplement: Additional file 1: — Multilingual abstracts in the six official working languages of the United Nations. [file 40249_2015_46_MOESM1_ESM.pdf]

Translation of the abstract into the six official working languages of the United Nations

تحديد بروفيل الاستجابة المناعية للخلايا البائية والتائية في حالات العدوى المشتركة بالسل الرئوي والدودة الشّصّية لدى الانسان

Xin-Xu Li, Jia-Xu Chen, Li-Xia Wang, Jun Sun, Shao-Hong Chen, Jun-Hu Chen, Xiao-Yan Zhang, Xiao-Nong Zhou

موجز

الخلفية: تلعب الاستجابات المناعية الخلطية والخلوية دورا واقيا من عدوى المتفطرة السلية (*Mycobacterium tuberculosis*). ولكن عدوى الدودة الشّصّية (hookworm) تنقص الاستجابة المناعية تجاه الدودة الشّصّية والمستضدات غير المشاركة في الحدث. حاليا، ما يزال من غير المعروف حدوث استجابة مناعية تجاه العدوى المشتركة بالمتفطرة السلية والدودة الشّصّية، على الرغم من أن العدوى المشتركة تشكل واحدة من مشاكل الصحة العامة في المناطق التي يستوطنها كل من السل الرئوي (PTB) والإصابة بالدودة الشّصّية. لذا من الضروري تقييم الاستجابات المناعية بالخلايا البائية والخلايا التائية تجاه العدوى المشتركة.

الطرق: في هذه الدراسة، تم إدراج سبع عشرة حالة مصابة بالسل الرئوي وبعدوى مشاركة بالدودة الشّصّية، ستا وعشرين حالة بالسل الرئوي، خمسة عشر مريضا بعدوى الدودة الشّصّية، وأربعة وعشرين فردا سليما للمراقبة دون إصابة بالسل الرئوي أو بالدودة الشّصّية. تم تقييم تعابير الواسمات الالتهابية CD3، CD4، CD8، CD10، CD19، CD20، CD21، CD25، CD27، CD38، FoxP3، و PD-1 على كل من الخلايا الفرعية البائية والتائية باستعمال قياس التدفق الخلوي متعدد الألوان.

النتائج: بالنسبة لتقسيمات الخلايا البائية ($CD19^+$)، فقد كانت الخلايا البائية التي لم يسبق تعرضها ($CD10^-CD27^-CD21^+CD20^+$)، الخلايا المصلية ($CD10^-CD27^+CD21^-CD20^-$)، وخلايا الذاكرة البائية المشابهة للنسج ($CD10^-CD27^-CD21^-CD20^+$) أعلى نسبة، في حين كان خلايا الذاكرة البائية المتبقية ($CD10^-CD27^+CD21^+CD20^+$) أقل نسبة في المجموعة المصابة بالعدوى المشتركة بالسل الرئوي والدودة الشّصّية مقارنة بالمجموعات الأخرى. لم تتغير تواترات خلايا الذاكرة البائية المفعلة ($CD10^-CD27^+CD21^-CD20^+$) بين المجموعات الأربعة. بالنسبة للخلايا الفرعية التائية ($CD3^+$)، كانت تواترات الخلايا التائية التنظيمية ($CD4^+CD25^+Foxp3^+$) والخلايا التائية $CD4^+$ و$CD8^+$ المنهكة ($CD4^+PD\text{-}1^+$ و $CD8^+PD\text{-}1^+$) هي الأعلى، وتواترات الخلايا التائية المفعلة $CD4^+$ و$CD8^+$ ($CD4^+CD38^+$ و$CD8^+CD38^+$) هي الأقل في المجموعة المصابة بالعدوى المشتركة مقارنة بالمجموعات الأخرى.

الاستنتاج: تم تحديد الأنماط المتغيرة للبروفيل الخلوي للمفاويات الجوالة لدى الأفراد المصابين بالعدوى المشتركة بالسل الرئوي والدودة الشّصّية، مما قد يشير إلى أن الاستجابات المناعية الخلطية والخلوية أكثر تثبطا.

Translated from English version into Arabic by Lina SM, through

TRANSLATORS WITHOUT BORDERS

结核菌和钩虫在人体内发生双重感染时 B 和 T 淋巴细胞的免疫反应

李新旭，陈家旭，王黎霞，孙俊，陈韶红，陈军虎，张晓燕，周晓农

摘要

引言：体液免疫和细胞免疫在结核菌感染时对宿主机体发挥着保护性作用，但钩虫感染可降低宿主对钩虫和其他同时存在的病原体的免疫反应。当前，在肺结核和钩虫病双重流行地区，结核菌和钩虫双重感染已经成为当地的公共卫生问题之一，但是双重感染时宿主机体免疫反应的变化情况尚不清楚，需要对其进行研究。

方法：选择了 17 个感染钩虫的肺结核患者、26 个未感染任何肠道寄生虫的肺结核患者、15 个仅感染钩虫的患者和 24 个未感染任何肠道寄生虫的健康对照，利用多色流式细胞术对所选研究对象外周血中 CD3、CD4、CD8、CD10、CD19、CD20、CD21、CD25、CD27、CD38、FoxP3 和 PD-1 的表达进行了检测。

结果：对于感染钩虫的肺结核患者，其 B 淋巴细胞亚群（$CD19^+$）中的幼稚 B 细胞（$CD10^-CD27^-CD21^+CD20^+$）、浆细胞（$CD10^-CD27^+CD21^-CD20^-$）和组织样记忆 B 细胞（$CD10^-CD27^-CD21^-CD20^+$）的比例较其他组高，静息记忆 B 细胞（$CD10^-CD27^+CD21^+CD20^+$）的比例较其他组低，而活化的记忆 B 细胞（$CD10^-CD27^+CD21^-CD20^+$）的比例在各组之间无差异；其 T 淋巴细胞亚群（$CD3^+$）中的调节性 T 细胞（$CD4^+CD25^+Foxp3^+$）、耗竭性 $CD4^+$T 细胞（$CD4^+PD\text{-}1^+$）和耗竭性 $CD8^+$T 细胞（$CD8^+PD\text{-}1^+$）的比例较其他组高，活化的 $CD4^+$T 细胞（$CD4^+CD38^+$）和活化的 $CD8^+$T 细胞（$CD8^+CD38^+$）的比例较其

他组低。

结论：在结核菌和钩虫双重感染时宿主机体的外周淋巴细胞分布比例发生了改变，显示体液免疫反应和细胞免疫反应均可能受到更多抑制。

Translated from English version into Chinese by LI Xin-xu

Profilage des réponses immunitaires des cellules B et T de l'organisme humain à la co-infection virale bactérienne bacille de Koch (*Mycobacterium tuberculosis*) et ankylostome.

Xin-Xu Li, Jia-Xu Chen, Li-Xia Wang, Jun Sun, Shao-Hong Chen, Jun-Hu Chen, Xiao-Yan Zhang, Xiao-Nong Zhou

Résumé

Contexte : Les réactions immunes humorales et cellulaires protègent l'organisme contre l'infection par la bactérie *Mycobacterium tuberculosis* (BK). Seulement, l'ankylostomiase réduit la réaction immunitaire liée aux antigènes d'ankylostome et aux antigènes spectateurs. À ce jour, les réponses immunitaires à la co-infection BK-ankylostome sont encore inconnues, bien que cette co-infection soit l'un des problèmes de santé publique dans les régions où la tuberculose pulmonaire (PTB) et l'ankylostomiase sont co-endémiques. Par conséquent, il est essentiel d'évaluer les réponses immunitaires des cellules B et T à cette co-infection.

Matériel et Méthodes : Dix-sept cas de co-infection PTB-ankylostome, 26 cas de PTB, 15 patients atteints d'ankylostomiase et 24 sujets témoins sains sans PTB ni ankylostomiase ont été mobilisés dans le cadre de cette étude. L'expression de CD3, CD4, CD8, CD10, CD19, CD20, CD21, CD25, CD27, CD38, FoxP3 et PD-1 sur les sous-ensembles des cellules B et T a été évaluée par cytométrie en flux avec marquage multicolore.

Résultats : Pour les sous-ensembles de la cellule B ($CD19^+$), les cellules B immatures ($CD10^-CD27^-CD21^+CD20^+$), les cellules plasmatiques ($CD10^-CD27^+CD21^-CD20^-$) et les cellules B mémoire semblables à des tissus ($CD10^-CD27^-CD21^-CD20^+$) avaient des proportions plus élevées, tandis que les cellules B mémoire au repos ($CD10^-CD27^+CD21^+CD20^+$) avaient des proportions plus faibles dans le groupe co-infecté par le BK et l'ankylostome par rapport aux autres groupes. Les fréquences des cellules B mémoire activées ($CD10^-CD27^+CD21^-CD20^+$) ne variaient pas dans les quatre groupes. Pour les sous-ensembles de la cellule T ($CD3^+$), les fréquences des cellules T régulatrices ($CD4^+CD25^+Foxp3^+$) et des cellules $CD4^+$ et $CD8^+$ T montrant un épuisement ($CD4^+PD\text{-}1^+$ et $CD8^+PD\text{-}1^+$) étaient plus élevées, et les fréquences des cellules $CD4^+$ et $CD8^+$ T activées ($CD4^+CD38^+$ et $CD8^+CD38^+$) étaient inférieures dans le groupe co-infecté par le BK et l'ankylostome par rapport aux autres groupes.

Conclusion : Les paramètres de changement du profil cellulaire des lymphocytes circulants ont été indentifiés dans les cas de co-infection BK-ankylostome de l'organisme humain, ce qui pourrait signifier que les réactions immunes humorales et cellulaires sont plus comprimées.

Translated from English version into French by emaleu, through

TRANSLATORS
WITHOUT BORDERS

Анализ иммунной реакции В-клеток и Т-клеток на коинфицирование микобактериями туберкулеза (*Mycobacterium tuberculosis)* и анкилостомами у человека

Ксин-Ксу Ли, Джиа-Ксу Чен, Ли-Ксиа Ванг, Юн Сун, Шао-Хонг Чен, Юн-Ху Чен, Ксяо-Ян Жанг, Ксяо-Нонг Жу

Реферат

История вопроса: Гуморальная и клеточная иммунная реакции играют роль защиты от инфицирования *микобактериями туберкулеза* (МБТ). Однако, анкилостомоз снижает иммунный ответ на анкилостомы и неспецифические антигены. В настоящее время иммунная реакция на коинфицирование МБТ и анкилостомами все еще остается неизвестной, хотя коинфицирование и является одной из проблем здравоохранения в эндемических областях туберкулеза легких и анкилостомоза. Следовательно, крайне необходимо оценить иммунную реакцию В и Т-клеток на коинфицирование.

Методы: В исследовании участвовали 17 больных с туберкулезом легких, коинфицированных анкилостомой, 26 больных с туберкулезом легких, 15 пациентов с анкилостомозом и 24 здоровых участника контрольной группы без туберкулеза легких или анкилостомоза. Оценивалась экспрессия CD3, CD4, CD8, CD10, CD19, CD20, CD21, CD25, CD27, CD38, FoxP3 и PD-1 в субпопуляциях В и Т-клеток с помощью многоцветной проточной цитометрии.

Результаты: В субпопуляциях В-клеток ($CD19^{+}$) наблюдались более высокие пропорции необученных В-клеток ($CD10^{-}CD27^{-}CD21^{+}CD20^{+}$), плазматических клеток ($CD10^{-}CD27^{+}CD21^{-}CD20^{-}$) и В-клеток памяти тканевого типа ($CD10^{-}CD27^{-}CD21^{-}CD20^{+}$), тогда как остающиеся В-клетки памяти ($CD10^{-}CD27^{+}CD21^{+}CD20^{+}$) содержались в более низкой пропорции в группе, коинфицированной МБТ и анкилостомой, по сравнению с другими группами. Частота активированных В-клеток памяти ($CD10^{-}CD27^{+}CD21^{-}CD20^{+}$) была одинаковой в четырех группах. В субпопуляциях Т-клеток ($CD3^{+}$) частота регуляторных Т-лимфоцитов ($CD4^{+}CD25^{+}Foxp3^{+}$) и истощенных Т-клеток $CD4^{+}$ и $CD8^{+}$ Т ($CD4^{+}PD\text{-}1^{+}$ и $CD8^{+}PD\text{-}1^{+}$) была выше, а частота активированных Т-клеток $CD4^{+}$ и $CD8^{+}$ ($CD4^{+}CD38^{+}$ и $CD8^{+}CD38^{+}$) была ниже в коинфицированной группе по сравнению с другими группами.

Выводы: Была определена модель изменения клеточного профиля циркулирующих лимфоцитов при коинфицировании человека МБТ и анкилостомами, которая может указывать на более высокую супрессию гуморальной и клеточной иммунной реакции.

Translated from English version into Russian by Alena Hrybouskaya, through

TRANSLATORS
WITHOUT BORDERS

Caracterización de las respuestas inmunitarias de células B y T a la coinfección de *Mycobacterium tuberculosis* y anquilostoma en humanos

Xin-Xu Li, Jia-Xu Chen, Li-Xia Wang, Jun Sun, Shao-Hong Chen, Jun-Hu Chen, Xiao-Yan Zhang, Xiao-Nong Zhou

Resumen

Información de referencia: Las respuestas inmunitarias humorales y celulares desempeñan un papel protector frente a la infección de *Mycobacterium tuberculosis* (MTB). No obstante, la infección de anquilostoma reduce la respuesta inmunitaria al anquilostoma y a otros antígenos. En la actualidad se siguen desconociendo las respuestas inmunitarias a la coinfección de MTB y anquilostoma, aunque la coinfección ha sido uno de los problemas de salud pública en áreas coendémicas de tuberculosis pulmonar y anquilostomosis. Por tanto es fundamental evaluar las respuestas inmunitarias de células B y T a la coinfección.

Métodos: En el estudio se registraron diecisiete casos de tuberculosis pulmonar coinfectados con anquilostoma,
26 casos de tuberculosis pulmonar, 15 pacientes con anquilostomosis y 24 controles de pacientes sanos sin tuberculosis pulmonar ni

anquilostomosis. Se evaluaron las manifestaciones de CD3, CD4, CD8, CD10, CD19, CD20, CD21, CD25, CD27, CD38, FoxP3 y PD-1 en subconjuntos de células B y T mediante citometría de flujo multicolor.

Resultados: Para los subconjuntos de células B ($CD19^+$), las células B naïf ($CD10^-CD27^-CD21^+CD20^+$), células plasmáticas ($CD10^-CD27^+CD21^-CD20^-$) y células B de memoria de tipo tejido ($CD10^-CD27^-CD21^-CD20^+$) presentaron proporciones más elevadas, mientras que las restantes células B ($CD10^-CD27^+CD21^+CD20^+$) presentaron proporciones más bajas en el grupo coinfectado con MTB y anquilostoma si se compara con otros grupos. Las frecuencias de células B de memoria activadas ($CD10^-CD27^+CD21^-CD20^+$) no diferían entre los cuatro grupos. Para los subconjuntos de células T ($CD3^+$), las frecuencias de células T reguladoras ($CD4^+CD25^+Foxp3^+$) y células T $CD4^+$ y $CD8^+$ agotadas ($CD4^+PD\text{-}1^+$ y $CD8^+PD\text{-}1^+$) eran más elevadas, y las frecuencias de células T $CD4^+$ y $CD8^+$ activadas ($CD4^+CD38^+$ y $CD8^+CD38^+$) eran más bajas en el grupo coinfectado si se compara con los otros grupos.

Conclusión: Se identificaron los patrones de variación del perfil celular de los linfocitos circulantes en coinfección humana de MTB y anquilostoma, los cuales podrían indicar que las respuestas inmunitarias humoral y celular se ven reprimidas.

Translated from English version into Spanish by Sergio Lorenzi, through

TRANSLATORS
WITHOUT BORDERS
